# Volumetric glutamate imaging (GluCEST) using 7T MRI can lateralize nonlesional temporal lobe epilepsy: A preliminary study

**DOI:** 10.1002/brb3.2134

**Published:** 2021-07-13

**Authors:** Peter N. Hadar, Lohith G. Kini, Ravi Prakash Reddy Nanga, Russell T. Shinohara, Stephanie H. Chen, Preya Shah, Laura E. M. Wisse, Mark A. Elliott, Hari Hariharan, Ravinder Reddy, John A. Detre, Joel M. Stein, Sandhitsu Das, Kathryn A. Davis

**Affiliations:** ^1^ Penn Epilepsy Center Department of Neurology Hospital of the University of Pennsylvania Philadelphia PA USA; ^2^ Department of Bioengineering University of Pennsylvania Philadelphia PA USA; ^3^ Center for Magnetic Resonance & Optical Imaging Department of Radiology University of Pennsylvania Philadelphia PA USA; ^4^ Department of Biostatistics, Epidemiology, and Informatics University of Pennsylvania Philadelphia PA USA; ^5^ Penn Image Computing & Science Lab University of Pennsylvania Philadelphia PA USA; ^6^ Department of Radiology University of Pennsylvania Hospital of the University of Pennsylvania Philadelphia PA USA

**Keywords:** epilepsy, glutamate imaging, MRI

## Abstract

**Introduction:**

Drug‐resistant epilepsy patients show worse outcomes after resection when standard neuroimaging is nonlesional, which occurs in one‐third of patients. In prior work, we employed 2‐D glutamate imaging, Glutamate Chemical Exchange Saturation Transfer (GluCEST), to lateralize seizure onset in nonlesional temporal lobe epilepsy (TLE) based on increased ipsilateral GluCEST signal in the total hippocampus and hippocampal head. We present a significant advancement to single‐slice GluCEST imaging, allowing for three‐dimensional analysis of brain glutamate networks.

**Methods:**

The study population consisted of four MRI‐negative, nonlesional TLE patients (two male, two female) with electrographically identified left temporal onset seizures. Imaging was conducted on a Siemens 7T MRI scanner using the CEST method for glutamate, while the advanced normalization tools (ANTs) pipeline and the Automated Segmentation of the Hippocampal Subfields (ASHS) method were employed for image analysis.

**Results:**

Volumetric GluCEST imaging was validated in four nonlesional TLE patients showing increased glutamate lateralized to the hippocampus of seizure onset (*p* = .048, with a difference among ipsilateral to contralateral GluCEST signal percentage ranging from −0.05 to 1.37), as well as increased GluCEST signal in the ipsilateral subiculum (*p* = .034, with a difference among ipsilateral to contralateral GluCEST signal ranging from 0.13 to 1.57).

**Conclusions:**

The ability of 3‐D, volumetric GluCEST to localize seizure onset down to the hippocampal subfield in nonlesional TLE is an improvement upon our previous 2‐D, single‐slice GluCEST method. Eventually, we hope to expand volumetric GluCEST to whole‐brain glutamate imaging, thus enabling noninvasive analysis of glutamate networks in epilepsy and potentially leading to improved clinical outcomes.

## Graphical abstract

Drug‐resistant epilepsy patients have worse outcomes after surgical resection when neuroimaging is nonlesional. Our prior work demonstrated the ability of 2‐D Glutamate Chemical Exchange Saturation Transfer (GluCEST) imaging to lateralize nonlesional temporal lobe epilepsy. We present a significant advancement to our previous single‐slice glutamate imaging with volumetric GluCEST, now enabling 3‐D analysis of glutamate networks and lateralization of hippocampal seizure onset in nonlesional temporal lobe epilepsy based on increased glutamate signal.

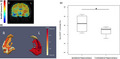

## 1 INTRODUCTION

In the one‐third of epilepsy patients who are medically refractory, approximately one‐half are diagnosed with temporal lobe epilepsy (TLE), often requiring surgical intervention to alleviate seizure burden (Davis et al., 2015). However, one‐third of refractory TLE patients have normal‐appearing MRIs and are two to three times more likely to have worse outcomes after surgery compared to those with lesions identified on MRI; this difference in surgical outcomes may be due to an inability to accurately localize the epileptogenic focus in the absence of structural MRI abnormalities (Carne et al., 2004; Téllez‐Zenteno et al., 2010).

Advances in imaging technology seek to address this problem (Kini et al., 2016). In combination with existing EEG and clinical variables, noninvasive imaging could vastly aid pre‐surgical planning, enabling more accurate resection of the seizure focus and better outcomes following surgery, especially in cases involving smaller lesions. 7T MRI holds the potential to better localize these lesions and detect previously undiscoverable abnormalities by providing higher resolution, superior contrast (T2*), and increased sensitivity to metabolites using magnetic resonance spectroscopy (MRS) or chemical exchange saturation transfer (CEST) (Krishnamoorthy et al., 2017; Thomas et al., 2008).

Prior evidence in human epilepsy patients and animal models using imaging and in‐vivo microdialysis suggests that glutamate concentrations are increased in seizure foci, likely reflecting impaired glutamate‐glutamine cycling in the setting of downregulation of astrocytic glutamine sythetase (Eid et al., 2013; Petroff et al., 2002; Wilson et al., 1996). MRS studies are less clear, at times indicating that glutamate is decreased in seizure foci. This variability is likely due to the poor spectral and spatial resolution inherent in the technique, with partial volume effects through the sulci, ventricles, or sclerotic tissue. CEST methods leverage chemical exchange between metabolites and bulk tissue water to achieve increased metabolite sensitivity and hence higher spatial resolution than MRS (Cai et al., 2012; Kogan, et al., 2013; Pfund et al., 2000; Simister et al., 2002).

We previously demonstrated that a single‐slice 2‐D Glutamate Chemical Exchange Saturation Transfer (GluCEST) imaging technique acquired using 7T MRI can lateralize and localize epileptogenic regions in the hippocampus, showing increased GluCEST signal, a measurement of glutamate concentration, in the ipsilateral total hippocampus and ipsilateral hippocampal head (Davis et al., 2015). While this work successfully demonstrated the feasibility of using GluCEST as a biomarker of epileptogenic tissue, a limitation of this early 2‐D CEST approach was a long acquisition time per slice, limiting the number of slices that could reasonably be acquired.

Three dimensional (3‐D) MRI methods provide improved efficiency for volumetric imaging as compared to multiple 2‐D acquisitions, particularly for methods such as CEST where much of the imaging time is devoted to magnetization preparation, which for GluCEST entails saturation transfer between glutamate's amide protons and bulk water. Here, we present initial confirmatory findings, using a novel 3‐D GluCEST imaging method, that demonstrate greater volumetric coverage at high spatial resolution of GluCEST signal changes in TLE patients (Krishnamoorthy et al., 2017).

## 2 METHODS

### 2.1 Subjects

Four patients with MRI‐negative TLE were recruited from the Penn Epilepsy Center (Table S1). All gave written consent, and the study was approved by the Institutional Review Board of the Hospital of the University of Pennsylvania.

### 2.2 7T GluCEST imaging

Imaging was performed on a Siemens 7 Tesla whole‐body MRI scanner equipped with a single transmit/32‐channel receiver array head coil. The MRI protocol included the following acquisitions: (a) A localizer scan, (b) T1‐w MPRAGE scan (TR/TI/TE = 2800/1500/4.4 ms, FA = 7^0^, GRAPPA=2, 170 sagittal slices, voxel size 0.8 mm^3^), (c) T2w‐MRI for hippocampal subfield segmentation (Yushkevich et al., 2016) (TR/TE =3000/388 ms; Matrix = 448 × 428; in‐plane resolution = 0.4 mm × 0.4 mm; slice thickness = 1.0 mm; 224 oblique coronal slices perpendicular to the long axis of the hippocampus), (d) B_0_ field‐map for EPI distortion correction sequence (TR/TE1/TE2 = 900/10/14 ms) and (e) 3D GluCEST coronal scan (TR/TE = 6/2.53 ms; 3D Matrix = 256 × 192 × 60; resolution = 1 mm^3^; GRAPPA = 2 × 1). Localized shimming was performed to keep B_0_ inhomogeneity to less than 0.5 p.p.m.

To acquire volumetric GluCEST, a partial 3‐D technique comprising of 60 1‐mm slices with a modified 3D FLASH sequence was used, employing a segmented elliptical center encoding strategy for the phase encode(ky) – slice encode(kz) plane (1‐mm isotropic voxel size). For GluCEST, a frequency selective saturation pulse train consisting of eight Hanning windowed pulses with a duration of 99.8 ms separated by 0.2 ms was used with a B_1rms_ of 3.06 µT. Similarly, for B0 mapping with water saturation shift referencing (WASSR), a frequency selective saturation pulse train consisting of two Hanning windowed pulses with a duration of 99.8 ms separated by 0.2 ms was used with a B_1rms_ of 0.29 µT (Kim et al., 2009). Raw GluCEST images were acquired by varying saturation offset frequencies from ±1.8 to ±4.2 p.p.m. with a step size of 0.3 p.p.m. and MT images were acquired with same parameters as above except that the saturation offset frequencies varied from ±20 to ±100 p.p.m. with a step size of 80 p.p.m. WASSR images were acquired by varying saturation offset frequencies from ±0 to ±1.2 p.p.m. with a step size of 0.1 p.p.m. For B_1_ map generation, hard pulses consisting of two predefined flip angles of 30 and 60 degrees were used for magnetization preparation. For B_1_ correction, two more CEST acquisitions were done as mentioned above at B_1rms_ of 2.04 µT and 1.02 µT in addition to the one at 3.06 µT, and these data will be used for B_1_ correction along with the B_1_ map as described elsewhere (Cai et al., 2012; Singh et al., 2013). Overall acquisition time for WASSR, B_1_mapping, GluCEST and MTR was ~45 min.

Voxel‐wise B0 estimation and correction were done based on the WASSR technique described elsewhere (Kim et al., Magn Reson Med 2009;61:1441–1450). To generate voxel estimates of percentage of GluCEST contrast, B_0_ corrected images were used, which is equal to 100 × (M_–3 ppm_−M_+3 ppm_)/M_‐3 ppm_, where M_–3 ppm_ and M_+3 ppm_ are B_0_ corrected images saturated at ∓3 ppm, relative to water (Cai et al., 2012).

### 2.3 Image analysis

7T MPRAGE images from all four patients were linearly registered to the respective CEST maps using Advanced Normalization Tools (ANTs) (Avants, Tustison, Song, et al., 2011). ASHS segmentation (an automated technique for segmenting hippocampal subfields and extra‐hippocampal medial temporal cortices from high‐resolution T2w‐MRI at 7T MRI) was then performed on 7T oblique coronal T2‐weighted imaging acquired perpendicular to the long axis of the hippocampus which resulted in segmentations of bilateral subfields of the hippocampi (Hadar et al., 2018; Shah et al., 2018). The segmentation included subiculum, Cornu Ammonis (CA) 1, 2, and 3 and dentate gyrus (DG). CA2 and CA3 were not included in the primary analyses because of the lower reliability of these regions, as opposed to the more reliable CA1, DG, and subiculum; this has been previously noted in the ASHS technique and is thought to be potentially due to inherent error and sampling issues involved in gross geometrical approximations of cellular transitions (Schoene‐Bake et al., 2014; Wisse et al., 2016). Overall, ASHS has been both pathologically and clinically validated (Hadar et al., 2018; Wisse et al., 2016). Supplementary analysis of the volumes was conducted on all hippocampal subfields.

These segmentations were warped to the patients' GluCEST images after compositely transforming segmentation from 7T coronal T2 space to 7T MPRAGE and finally to the patients' GluCEST space. In addition, ANTs DiReCT/Atropos cortical thickness pipeline was run on 7T MPRAGE images to generate three tissue segmentations of gray‐matter, white‐matter, and CSF for GluCEST measurements of gray‐matter alone in the cerebral hemispheres, as well as of gray‐matter‐standardized (based on proportion) GluCEST signal of the hippocampal subfields in the supplementary analysis (Avants, et al., 2011; Das et al., 2009). Analysis of GluCEST values and volumes in different hippocampal regions was performed using in‐house Python software, and statistical analysis, involving two‐sample paired Student's *t*‐tests (given the measurement of multiple observations in a single patient), was conducted using R.

## 3 RESULTS

**FIGURE 1:**
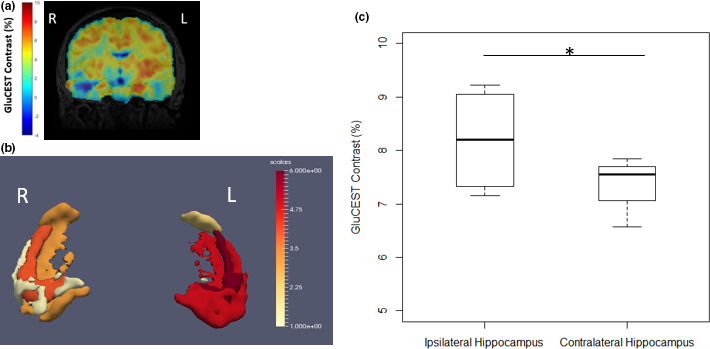
Increased 3‐D GluCEST signal in the ipsilateral hippocampus in a 26‐year‐old with MRI‐negative left temporal lobe epilepsy. (a) Coronal slice of full‐brain GluCEST Map registered to MPRAGE. All coronal slices were averaged across 5 voxels in each slice to improve SNR and to mimic a thick slab (5 mm) from the 2D sequence, with GluCEST contrast percentage scaling from −4 (blue) to 10 (red). (b) After masking the GluCEST map to the hippocampus using T2‐based ASHS hippocampal segmentation, the GluCEST signal in each voxel, averaged across all 4 patients, was averaged into 6‐quantiles (for better visualization) across hippocampal and extrahippocampal regions of interest, from 1 (yellow) to 6 (red). These were then reconstructed using the Paraview imaging software and scaled appropriately. (c) In 4 left‐sided MRI‐negative temporal lobe epilepsy patients, we see a statistically significant increased GluCEST signal in the ipsilateral (left) hippocampus (*p* =.048, 1‐tailed 2‐sample paired Student's *t* test, *n* = 4), indicating increased glutamate

In all four nonlesional (MRI‐negative) TLE patients, the GluCEST signal was increased in the ipsilateral total hippocampus relative to the contralateral total hippocampus using the 3‐D technique (*p* =.048, with a difference among ipsilateral to contralateral GluCEST signal ranging from −0.05 to 1.37). Left‐sided lateralization is clearly seen in the representative image of a single patient in a coronal slice of the total GluCEST map (Figure 1a) and a 3‐D representation (Figure 1b). A graphical representation of the GluCEST signal averaged across the ipsilateral and contralateral hippocampi of all four patients, respectively, demonstrates the increased signal on the left, ipsilateral to seizure onset (Figure 1c).

This difference in GluCEST signal is likely due to the underlying chemical processes at play and not clear due to volumetric differences, with additional analysis of the hippocampal subfields providing further evidence of this. Table S2 analyzes the hippocampal subfields among the same patients and does not note any significant difference between the subfield volumes, while Table S3 notes that the gray‐matter standardization of the GluCEST signal approaches statistical significance with Bonferroni correction when comparing the left‐to‐right subiculum (6.82–5.41 at *p* =.02). This may point to the importance of gray‐matter in providing some of the GluCEST signal asymmetry in the subiculum noted in Table 1.

**TABLE 1:** Volumetric GluCEST findings in 4 left‐sided nonlesional TLE patients, comparing regions ipsilateral and contralateral to seizure onset

Bilateral regions tested	Two‐sample paired *t*‐test for means, comparing ipsilateral versus contralateral (one‐tailed)	GluCEST values ipsilateral (%), range of values	GluCEST values contralateral (%), range of values	Ipsilateral‐contralateral (%), range of values
Total hippocampus	*p* =.048*	7.15–9.22	6.57–7.85	−0.05–1.37
CA1	*p* =.153	7.12–8.80	6.76–8.41	−0.53–2.05
Dentate gyrus	*p* =.112	7.81–9.42	6.48–8.83	−0.50–2.02
Subiculum	*p* =.034*	6.00–9.86	5.88–9.00	0.13–1.57
Cerebral hemispheres	*p* = 0.814^a^	5.44–8.03	5.52–7.80	−0.23–0.24

^a^ Two‐tailed paired *t*‐test for means.

* Statistically significant for *p* <.05, *n* = 4.

As in our previous study, the ipsilateral hippocampus demonstrated a significantly higher GluCEST signal than did the contralateral side (Table 1); however, only the subiculum demonstrated similar lateralization (*p* = .034, with a difference among ipsilateral to contralateral GluCEST signal ranging from 0.13 to 1.57). The other hippocampal subfields calculated in the primary analysis, namely CA1 (*p* = .153, with a difference among ipsilateral to contralateral GluCEST signal ranging from −0.53 to 2.05) and the dentate gyrus (*p* = .112, with a difference among ipsilateral to contralateral GluCEST signal ranging from −0.50–2.02) did not demonstrate similar lateralization. As a quantitative control for the GluCEST technique, comparison of the cerebral hemispheres indicated equivalent GluCEST on either side (*p* = .814), as would be expected in a TLE patient without extratemporal involvement.

Further analysis supporting the ipsilateral increase of the GluCEST signal is described in the [Link], [Link]. Figure S1 provides a visual depiction of all of the coronal slices of the total GluCEST map registered to MPRAGE all four patients, showing overall increased GluCEST signal on the left (ipsilateral). Figure S2 provides a graphical representation of the total hippocampus GluCEST signal in each patient.

## 4 DISCUSSION

### 4.1 Clinical significance

Our study demonstrates that a volumetric GluCEST imaging technique can accurately lateralize seizure foci in nonlesional TLE patients. Although this capability was only tested in a small cohort of four patients, our findings demonstrate significantly higher GluCEST signal in the ipsilateral total hippocampus compared to that of the contralateral side. This also confirms our prior observations in a different set of four nonlesional TLE patients using a 2‐D GluCEST imaging technique. Moreover, the ipsilateral subiculum showed significantly higher GluCEST signal, while the ipsilateral CA1 and ipsilateral dentate gyrus demonstrated higher GluCEST signal as well, albeit not statistically significant.

While our previous 2‐D GluCEST study did not perform subfield segmentation of the hippocampus, it indicated increased GluCEST signal in the ipsilateral total hippocampus and the hippocampal head, which approximately mimics our results in more detailed sub‐volumes (Davis et al., 2015). Although an accurate comparison between studies is limited given a different patient group and dimension increase, the GluCEST signal appears similar. For 2‐D GluCEST, the GluCEST signal of the total hippocampi ranged from 8.69 to 11.16 ipsilaterally, 7.41 to 10.32 contralaterally, and a difference of 0.46 to 1.28. For our volumetric study, the differences in GluCEST signal of the total hippocampi are similar: 7.15–9.22 ipsilaterally, 6.57–7.85 contralaterally, and a difference of −0.05–1.37.

The limited subfield differentiation by GluCEST signal seen in this study is likely due to the variations from the small sample size, as well as the 1mm isotropic voxel size used in the CEST imaging technique. While it is possible that this multi‐slice study is accurately indicating an increased glutamate signal in the ipsilateral hippocampus, and especially in the ipsilateral subiculum, the previous study did not investigate hippocampal subfields and thus cannot provide a complete comparison; however, existing literature has suggested increased glutamate primarily in CA1‐3 (Eid et al., 2004, 2007, 2013; Petroff et al., 2002). Indeed, CA1 showed an increased GluCEST signal in this study, though not statistically significant in this small sample. Further work with larger sample sizes will help us gain a greater understanding of the role and extent of glutamate in the hippocampus and its subfields.

Our findings speak to the importance and potentially wide application of a whole‐brain volumetric GluCEST technique. Glutamate is a key neurotransmitter involved not only in epilepsy but also in other spine and brain disorders, like multiple sclerosis and psychosis (Kogan, Singh, et al., 2013; Roalf et al., 2017). GluCEST has two orders of magnitude higher sensitivity than MRS for detecting glutamate with a higher spatial resolution, which can potentially allow for a better understanding and diagnosis of neurologic and psychiatric disorders (Cai et al., 2012). A downside to this approach is the sensitivity to B0 inhomogeneities that require longer scan times, but recent advances in a deep learning applications can reduce GluCEST imaging time and improve signal‐to‐noise ratio, thus paving the way for the whole‐brain approach (Li et al., 2020). While 2D techniques can provide some information on underlying excitatory processes, a whole‐brain volumetric GluCEST approach can delineate entire glutamate networks involved in neurologic function and dysfunction with an eye toward clinical application.

Our advance from single‐slice to volumetric imaging is a stepping stone toward eventual whole‐brain, volumetric GluCEST imaging, which could allow for localization of seizure onset and mapping of the epileptic network with greater accuracy in nonlesional and neocortical epilepsy patients. Such an approach could also facilitate better guidance for intracranial electroencephalography (EEG) implantation and neuromodulation, as well as potentially contribute to surgical planning, thus resulting in improved post‐surgical outcomes.

### 4.2 Limitations

Although volumetric glutamate imaging is a significant step forward from the previous 2‐D technique, there are several limitations to this study: small sample size, limited subfield glutamate localization, and the need for further optimization of the B_1_ correction strategy in the 3‐D GluCEST method in the neocortex.

The 2D GluCEST protocol was not performed on these patients since this was a feasibility study. As a result, the volumetric GluCEST results determined in this study were compared to those of the prior 2D GluCEST study. Our future whole‐brain volumetric GluCEST study will compare the 2D, volumetric, and whole‐brain volumetric approaches.

As a preliminary study aiming to eventually apply GluCEST volumetrically across the whole brain, controls were not scanned, which can limit some of the applicability of our findings. Additionally, as a result of the nonlesional, lateralized patient population, only left‐sided TLE patients could be analyzed. Future analysis will include both controls, right‐sided TLE, and lesional epilepsy patients as well.

The inclusion of patients with noted lateralized PET hypometabolism may have also introduced some degree of bias, demonstrating some localization on an imaging biomarker. However, PET is not always universally pursued in a pre‐surgical workup, so the mix of PET findings is likely consistent with clinical presentations. Ongoing work by our lab seeks to incorporate a multi‐modality imaging biomarker approach to pre‐surgical epilepsy decision‐making, in which PET and eventually GluCEST will play important roles (Kini et al., 2021).

### 4.3 Future directions

Further developmental work is needed to expand the volumetric methodology to provide whole‐brain coverage, with optimal B_1_ correction algorithms within a clinically viable scan time. Work along these lines is currently ongoing. Following such developments, we aim to further investigate the role of glutamate in hippocampal subfields and correlate these findings to electrophysiological and clinical outcomes, and to develop a greater understanding of epilepsy excitatory networks, particularly in nonlesional, neocortical epilepsy.

## CONFLICT OF INTEREST

R.R. and H.H. hold the patent (U.S. 20120019245 A1) on CEST MRI methods for imaging metabolites and the use of these as biomarkers.

## AUTHOR CONTRIBUTION

KAD, JAD, and RR contributed to conceptual development of the study. KAD developed the clinical protocol and recruited the subjects. KAD, PNH, and LGK determined the analysis of data. JAD, RPRN, ME, HH, and RR developed the GluCEST technique. JMS read the PET and GluCEST scans. SD, PS, and LEMW developed the segmentation. LGK and SD processed the data. PNH wrote the manuscript. All authors contributed to the final manuscript.

### PEER REVIEW

The peer review history for this article is available at https://publons.com/publon/10.1002/brb3.2134.

## Supporting information

App S1

App S2

## Data Availability

The data that support the findings of this study are available from the corresponding author upon reasonable request.
