# Supplementary material for: Volumetric glutamate imaging (GluCEST) using 7T MRI can lateralize nonlesional temporal lobe epilepsy: A preliminary study
Source: Brain Behav. 2021 Jul 13;11(8):e02134. doi: 10.1002/brb3.2134 (PMC8413808; doi:10.1002/brb3.2134)
Supplement: Supplementary file 1 — App S1 [file BRB3-11-e02134-s002.docx]

Appendix S1

## *Supplementary Table 1:* Patient Demographics.

| **Patient** | **Age** | **Gender** | **Clinical MRI** | **FDG-PET** | **Seizure Duration** | **Seizure Frequency** | **Seizure Localization** |
| --- | --- | --- | --- | --- | --- | --- | --- |
| A | 26 | Male | Normal | None | 6 years | 2-3 CPS & GTC/year | Left Temporal (scalp EEG) |
| B | 39 | Male | Left cerebellar developmental venous anomaly | Mild left anterior medial temporal hypometabolism | 4 years | 3-7 SPS/week | Left temporal (scalp EEG) |
| C | 32 | Female | Normal | Normal | 3 years | CPS daily, 1 GTC/month | Left temporal (scalp EEG) |
| D | 45 | Female | Small, nonspecific periventricular T2 hyperintensities | Left anterior temporal hypometabolism | 21 years | 2-4 SPS/week, 1 CPS/week | Left temporal (intracranial EEG)* |

*Underwent left temporal lobectomy.

SPS, simple partial seizure; CPS, complex partial seizures; GTC, generalized tonic-clonic seizure.

*Supplementary Table 2:* Hippocampal Subfield Volume Analysis

| *Region* | *Left volume^1^* | *Right volume^1^* | *p-Value^2^* |
| --- | --- | --- | --- |
| CA1 | 691 ± 166 | 684 ± 157 | 0.49 |
| CA2 | 16.8 ± 5.8 | 23.8 ± 10.0 | 0.28 |
| CA3 | 90.8 ± 28.5 | 69.8 ± 14.8 | 0.08 |
| DG | 542 ± 129 | 709 ± 161 | 0.028 |
| Subiculum | 770 ± 145 | 817 ± 145 | 0.27 |
| *^1^ Mean ± standard deviation. ^2^ 2-tailed paired t-test. Significance with Bonferroni correction (p <0.01) indicated in bold.* | | | |

*Supplementary Table 3:* Gray Matter Analysis of GluCEST Signal

| *Region* | *Left % gray matter^1^* | *Right % gray matter^1^* | *p-Value^2^* | *Left standardized GLUCEST ^3^* | *Right standardized GLUCEST ^3^* | *p-Value^2^* |
| --- | --- | --- | --- | --- | --- | --- |
| CA1 | 95.8 ± 4.7 | 94.0 ± 4.2 | 0.79 | 7.63 ± 0.57 | 6.98 ± 0.85 | 0.35 |
| CA2 | 92.9 ± 8.1 | 95.7 ± 4.9 | 0.66 | 7.12 ± 0.81 | 7.14 ± 1.40 | 0.99 |
| CA3 | 91.4 ± 5.3 | 95.2 ± 2.7 | 0.12 | 6.83 ± 0.64 | 6.16 ± 1.25 | 0.28 |
| DG | 95.5 ± 5.3 | 96.0 ± 2.7 | 0.73 | 8.13 ± 0.77 | 7.35 ± 0.96 | 0.22 |
| Subiculum | 84.9 ± 8.0 | 75.2 ± 8.6 | **0.008** | 6.82 ± 0.98 | 5.41 ± 0.48 | 0.02 |
| *^1^ Mean ± standard deviation. ^2^ 2-tailed paired t-test comparing left versus right. Significance with Bonferroni correction (p <0.01) indicated in bold. ^3^ Mean ± standard deviation of GluCEST signal standardized to gray matter percentage.* | | | | | | |
