# Supplementary material for: Volumetric glutamate imaging (GluCEST) using 7T MRI can lateralize nonlesional temporal lobe epilepsy: A preliminary study
Source: Brain Behav. 2021 Jul 13;11(8):e02134. doi: 10.1002/brb3.2134 (PMC8413808; doi:10.1002/brb3.2134)
Supplement: Supplementary file 2 — App S2 [file BRB3-11-e02134-s001.docx]

Appendix S2

*Supplementary Figure 1:* Visual depiction of increased 3-D GluCEST signal in the ipsilateral hippocampi of 4 patients with MRI-negative left temporal lobe epilepsy.


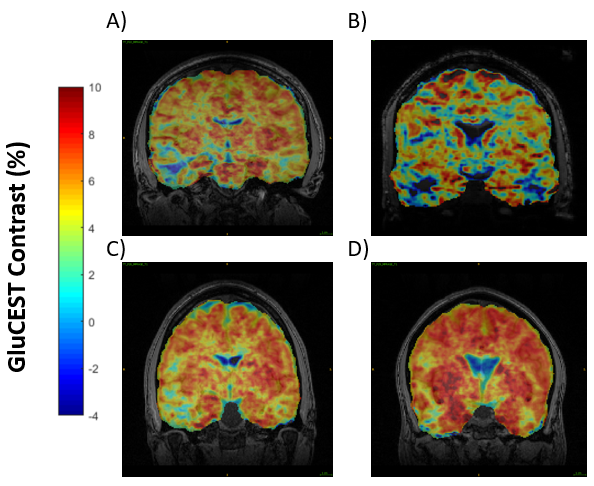


Coronal slices of full-brain GluCEST map registered to MPRAGE. All coronal slices were averaged across 5 voxels in each slice to improve SNR and to mimic a thick slab (5 mm) from the 2D sequence, with GluCEST contrast percentage scaling from -4 (blue) to 10 (red). A) Patient A: 26-year-old male. B) Patient B: 39-year-old male. C) Patient C: 32-year-old female. D) Patient D: 45-year-old female.

*Supplementary Figure 2:* Graphical depiction of increased GluCEST signal in the ipsilateral hippocampi of 4 patients with MRI-negative left temporal lobe epilepsy.

Increased GluCEST signal is seen in the left hippocampi of 4 patients, corresponding to the region ipsilateral to seizure onset. Patient A is a 26-year-old male, patient B is a 39-year-old male, patient C is a 32-year-old female, and patient D is a 45-year-old female.
